# Transcriptome profiling of a synergistic volumetric muscle loss repair strategy

**DOI:** 10.1186/s12891-023-06401-1

**Published:** 2023-04-24

**Authors:** Kevin Roberts, John Taehwan Kim, Tai Huynh, Jacob Schluns, Grady Dunlap, Jamie Hestekin, Jeffrey C Wolchok

**Affiliations:** 1 Cell & Molecular Biology Program, University of Arkansas Fayetteville, Arkansas, USA; 2 Department of Biomedical Engineering, University of Arkansas Fayetteville, Arkansas, USA; 3 Ralph E. Martin Department of Chemical Engineering, University of Arkansas Fayetteville, Arkansas, USA

**Keywords:** Muscle injury and repair, Volumeric muscle loss, Biomaterial implant, Transcriptomic profiling, Pre-clinical animal model research

## Abstract

Volumetric muscle loss overwhelms skeletal muscle’s ordinarily capable regenerative machinery, resulting in severe functional deficits that have defied clinical repair strategies. In this manuscript we pair the early in vivo functional response induced by differing volumetric muscle loss tissue engineering repair strategies that are broadly representative of those explored by the field (scaffold alone, cells alone, or scaffold + cells) to the transcriptomic response induced by each intervention. We demonstrate that an implant strategy comprising allogeneic decellularized skeletal muscle scaffolds seeded with autologous minced muscle cellular paste (scaffold + cells) mediates a pattern of increased expression for several genes known to play roles in axon guidance and peripheral neuroregeneration, as well as several other key genes related to inflammation, phagocytosis, and extracellular matrix regulation. The upregulation of several key genes in the presence of both implant components suggests a unique synergy between scaffolding and cells in the early period following intervention that is not seen when either scaffolds or cells are used in isolation; a finding that invites further exploration of the interactions that could have a positive impact on the treatment of volumetric muscle loss.

**Supplementary Information:**

The online version contains supplementary material available at 10.1186/s12891-023-06401-1.

## Background

Skeletal muscle exhibits an innate ability to regenerate small amounts of functional tissue following minor traumas including strains and contusions [1].The immediate response to mild muscle injury consists of partial necrosis of damaged myofibers and their degradation by neutrophils, followed by migration of macrophages to the injury which continue to degrade necrotic tissue [2]. Key cytokines released from injured muscle matrix promotes satellite cells residing between myofibers to reenter the cell cycle, proliferate, and migrate to sites of injury as myoblasts, after which they undergo fusion to form nascent myofibers [3–5]. Over the course of several days, the community of macrophages present will shift toward a range of phenotypes secreting signals promoting myoblast differentiation and connective tissue deposition by fibroblasts [6].

Alternatively, in the case of volumetric muscle loss (VML), satellite cell recruitment and differentiation are overwhelmed by inflammatory signaling and damage to the basal lamina, with ECM-deposition featuring prominently in the weeks following injury [7]. With the loss of critical native scaffolding, satellite cell migration is restricted [8]. In relatively mild muscle injuries, remnants of degenerated myofibers act as structural scaffolds for the bidirectional migration of satellite cells [9], but these are comparatively absent in VML. Consequently, VML injuries result in the loss of structural guidance for myogenesis and the deposition of non-contractile scar tissue which prevents the intrusion of nascent myofibers, contributing to a permanent deficit in muscle force.

Exploratory biomaterials for treatment of VML generally attempt to restore the architectural and compositional cues lost to injury and thereby provide a pro-regenerative substrate for endogenous progenitor cell migration. The more promising scaffolding materials appear to be those derived from native tissues, which can be remodeled by the body’s own wound healing machinery. This includes scaffolds prepared using decellularized skeletal muscle (DSM), porcine small intestinal, and bladder tissue. However, the use of tissue derived scaffolds on their own has yielded mixed results, with little evidence of de-novo muscle fiber regeneration [10–13]. To enhance muscle regeneration, synergistic therapies in which tissue scaffolds are supplemented with exogenous precursor cells have been explored [14–18]. Founded on the premise that the scaffold provides an enabling substrate for regeneration by co-delivered cells, these combinatorial (scaffold + cells) tissue engineering strategies have been able to restore approximately half of the contractile force lost to VML injury [19].

The mechanisms by which these combinatorial therapies (scaffold + cells) may enhance muscle recovery when compared to either strategy used in isolation remains unclear, particularly during the early weeks following injury. The lower level of recovery occurring with the use of scaffolds or cells alone emphasizes the importance of a multifactor approach to VML treatment, and suggests a biomolecular synergy exists between delivered cells and scaffolds which to date has not been explored in depth. Here we extend the body of VML knowledge by profiling the transcriptome-wide responses of VML-injured tissue to scaffolding and cell delivery strategies employed in isolation and in combination at 3 to 14 days post injury (DPI) to better assess early regulatory changes potentially mediating the significant differences observed in long-term functional outcome between these distinct tissue engineering schemes. Clarifying the effects of these therapies on the complex inflammatory, fibrotic, and regenerative networks in the early period following injury contributes to a foundational understanding of VML pathobiology upon which to further refine VML repair strategies for the clinic.

## Methods

### Animals

Sprague Dawley rats (pre-surgical mass ~ 350 g) were used in this study (3 animals/ group for 3 DPI cohort and 5 animals / group for 14 DPI cohort). The 3 and 14 day timepoints are supported by findings by Corona in which they observed a rise in many key regenerative markers during the first two weeks post VML injury and repair [20]. Buprenorphine (0.1 mL at 0.3 mg/mL) was administered to all rats subcutaneously every eight hours for postoperative analgesia and access to Rimadyl was provided at up to 1 mg per day for seven days post-injury. Food and water were provided *ad libitum*. All animal procedures were approved by and performed in accordance with the guidelines of the Institutional Animal Care and Use Committee of the University of Arkansas.

### Injury and repair

**Fig. 1:**
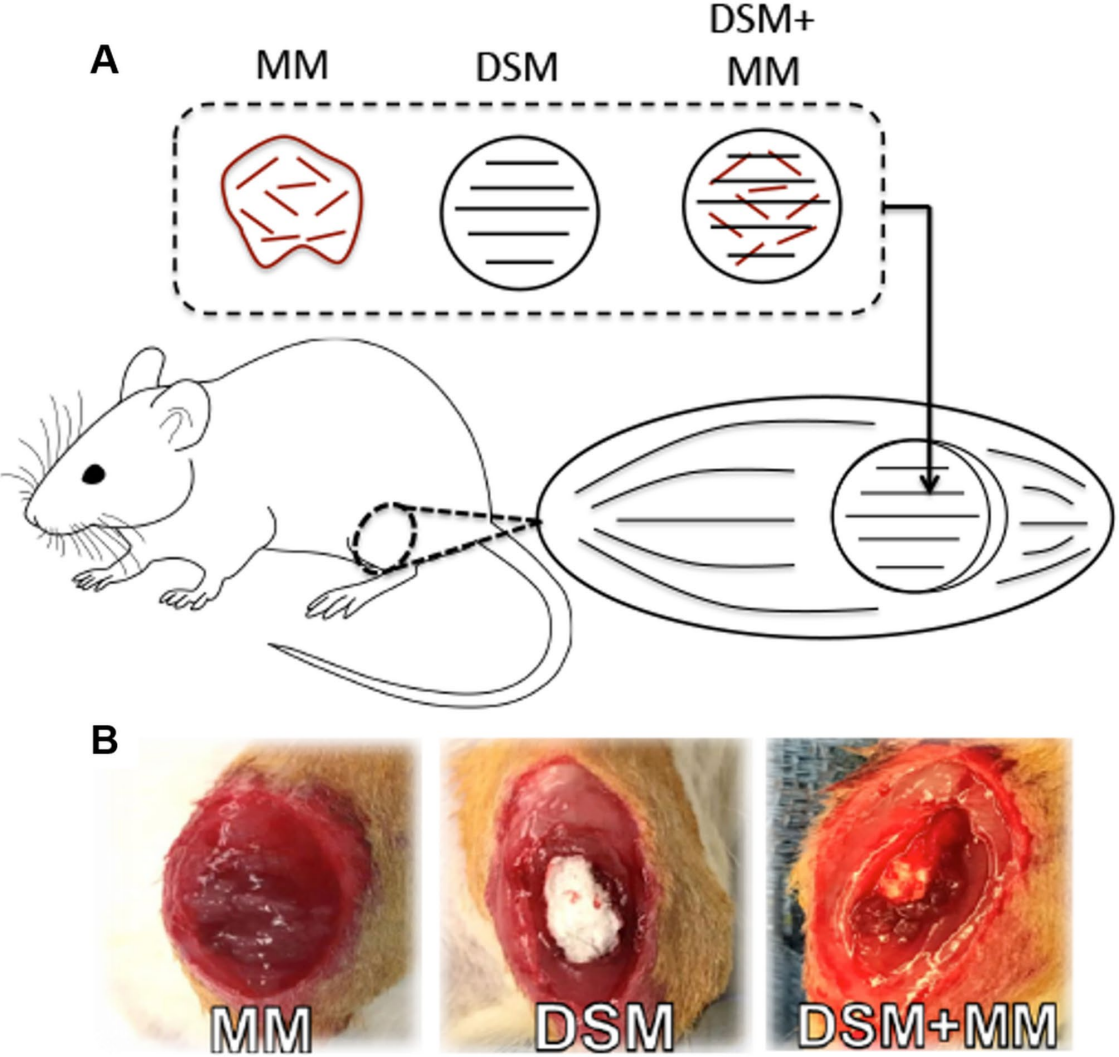
Creation of VML injuries and subsequent repair strategies (**A**) Rat tibialis anterior muscles were ablated with an 8 mm diameter biopsy punch to create a VML defect removing approximately 20% of muscle mass [57]. VML defects were immediately subjected to implant repair strategies (**A-B**) including minced muscle (MM), decellularized skeletal muscle (DSM), and a combination of the two (DSM + MM).

VML injuries were created in rats using an established VML model [21]. Briefly, a lateral incision was made on the lower left leg separating skin and fascia to expose the tibialis anterior (TA) muscle. An 8 mm biopsy punch was used to remove muscle tissue (~ 100 mg) from the middle third of the TA to a depth of 3 mm (Fig. 1A). Subsets of rats underwent repair of VML injury with autologous minced muscle grafts (MM), decellularized skeletal muscle prepared from rat tibialis anterior (DSM), and a combination of DSM and MM (DSM + MM), Fig. 1B). The use of MM, sourced from small portions of autologous donor muscle, is a clinically translatable source of muscle precursor cells that has shown promise by us and others [8, 22–26]. DSM was prepared by incubation of rat TA in 1% SDS for one week followed by treatment with 1kU/mL DNAse I and RNAse A. Constructs were incubated for eight hours in penicillin-streptomycin solution, lyophilized, and stored at -20 °C pending implantation. MM autografts were prepared during surgery by mincing 50% of the biopsied tissue using microscissors. Fascia and skin were closed using interrupted stitches with 6 − 0 Vicryl sutures.

### Functional assessment

Peak tetanic contractile force of injured and uninjured contralateral limbs was measured at three days and fourteen days post-injury as previously described [27]. Briefly, peroneal nerves of anesthetized rats (2–2.5% isoflurane) were stimulated (150 Hz, 0.1 ms pulse width, 400 ms pulse train) by an S88 pulse stimulator (Grass Technologies, West Warwick) to induce contraction of the tibialis anterior (TA) muscle. Measurement of raw force output (N) was enabled by securing the foot of the limb to a force transducer system (Aurora Scientific, Ontario) with surgical tape. Peak tetanic force for each limb was calculated by the mean of three contractions and normalized to animal mass (N/kg body weight). All animals were then euthanized by carbon dioxide inhalation in accordance with guidelines provided by the AVMA Panel on Euthanasia of Animals.

### RNA preparation and sequencing

Samples of TA muscles (n = 3 / group / timepoint) encapsulating the interface between the defect and defect-adjacent regions were harvested from euthanized rats at three days and fourteen days post-injury using a 2 mm biopsy punch, snap frozen on liquid nitrogen, and stored at -80 °C pending RNA isolation. Tissues were thawed and homogenized for 15 s at room temperature. Total RNA was isolated using the Purelink RNA Mini Kit (ThermoFisher). RNA concentration was determined by nanodrop spectrophotometry, with RNA quality (28 S/18s > 2, RIN > 7) confirmed using a Tapestation (Agilent Technologies). cDNA libraries were sequenced on the BGISeq-500 platform to a mean depth of 20,000,000 reads per library.

### RNA-seq data analysis

RNA sequencing reads were mapped to the *Rattus norvegicus* genome (RGSC build 6.0) using STAR [28]. Reads were quantified using FeatureCounts [29], followed by TMM count normalization and analysis of differential expression in edgeR [30]. Differential expression was classified using a false discovery rate (FDR) cutoff of 0.05 and a minimum fold change of 1.5. Visualization of group intersections for differential gene expression was performed using UpsetR [31]. Enrichment analysis of gene sets was performed against the Biological Process and Cellular Component Gene Ontology databases in ShinyGO [32]. Pathway level analysis was also performed using Ingenuity Pathway Analysis (Qiagen) [33].

### Statistics

Statistical analyses for assessment of muscle force and mass were performed using Prism 9 (Graphpad, La Jolla, California), using repeated measures ANOVA with post-hoc Dunnett’s test. Assessment of significance for differential gene expression was performed using EdgeR with TMM normalization of read counts and adjustment of p-values for multiple comparisons by the Benjamini-Hochberg procedure. Linear regression using the least squares method was used to model the relationship of Reln and Robo1 gene expression to peak contractile muscle torque for all treatment groups at 14 DPI. The quality of the relationship was evaluated with the Coefficient of Determination (R^2^) and the significance of the relationship was examined using ANOVA. Significance was accepted at P ≤ 0.05 (*) P ≤ 0.01 (**), P ≤ 0.001 (***), and P ≤ 0.0001 (****). Quantitative data are displayed as mean ± standard deviation.

### Data Availability

All RNA-sequencing FASTQ files and processed transcipts expression files were deposited in the Gene Expression Omnibus (https://www.ncbi.nlm.nih.gov/geo/) under accession number GSE125896. Supplementary Figures are publicly available at a GitHub repository (http://github.com/RobertsEng/VML-RNA-seq).

## Results

### Early functional outcomes of VML injury are not yet affected by repair strategies

**Fig. 2:**
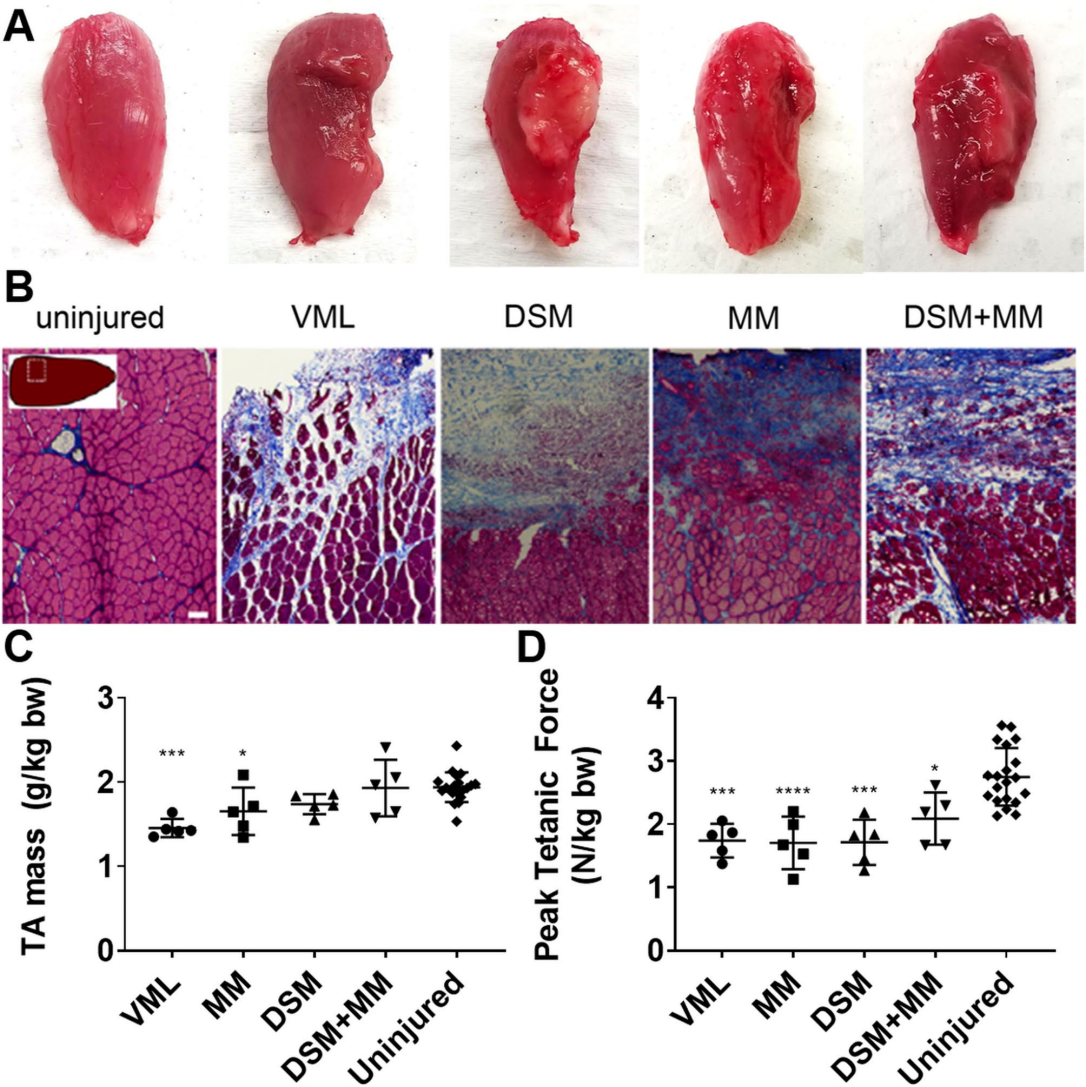
Muscle histology and electrophysiology data (**A**) Gross appearance of tibialis anterior muscles for groups at fourteen DPI. TA muscle cross-sections were stained with Masson’s Trichrome. (**B**) Representative 2-week uninjured, VML, DSM, MM and DSM + MM repair groups are presented. Magnified (100X) images are shown. Inset indicates approximate location of magnified image within the TA cross-section. Scale bar = 100 μm. (**C**) Tibialis anterior mass (g/kg rat body weight) and electrophysiological measurement of mean peak contractile force for all groups (**D**) at fourteen DPI, indicating deficits in functional outcome among VML, MM, DSM, and DSM + MM-treated muscles relative to the uninjured contralateral limb at 14 DPI (p = 0.0001, < 0.0001, < 0.0001 and 0.0127, respectively). The *, **, ***, and **** indicate statistically significant difference of p < 0.05, p < 0.01, p < 0.001, and p < 0.0001 when comparing each group to uninjured controls. Error bars are presented as ± standard deviation, with N = 4–5 animals per treatment group. Scale-bar = 1 cm

All animals tolerated the surgery well and reached the study endpoints. No significant differences in animal growth were observed. Ablation of the rat tibialis anterior (TA) resulted in defects clearly visible at 3 and 14 days post injury (DPI) (Fig. 2A). While no clear differences were noted in the gross appearance of VML and MM-treated muscles, DSM and DSM + MM implants remained intact in the site of the defect, with the DSM + MM appearing better integrated with the muscle than DSM alone (Fig. 2A-B). Masses of excised TA muscles were statistically indistinguishable from excised contralateral uninjured TA across all groups at 3 DPI (Supplemental Fig. 1). At 14 DPI, the mean mass of VML and MM-treated TA’s were significantly lower relative to uninjured TA (74.2% of uninjured TA mass with p = 0.0001 and 84.3% of uninjured TA mass with p = 0.0257, respectively), while DSM and DSM + MM exhibited on average 88.6% (p = 0.1579) and 98.4% (p = 0.9970) of uninjured muscle mass, respectively (Fig. 2C).

Unrepaired, repaired, and uninjured contralateral limbs were assessed for peak tetanic force output at both time points. At 3 DPI, MM and DSM + MM -treated groups exhibited significantly lower force output (N/kg body weight) than uninjured contralateral muscles (64.2% and 41.6% of uninjured limb force, p = 0.0415 and p = 0.0335, respectively) (Supplemental Fig. 1). Unrepaired, MM, DSM, and DSM + MM treatments exhibited significantly lower force output (N/kg body weight) at 14 DPI relative to uninjured muscles (63.3%, 62.1%, 62.3%, and 76.0% of uninjured limb force, p = 0.0001, p < 0.0001, p < 0.0001, and p = 0.0127, respectively) (Fig. 2D**)**. No significant differences in contractile kinematics were detected between groups during the first two weeks of recovery.

### Muscle transcriptomes vary sharply with repair strategy at two weeks post-injury

**Fig. 3:**
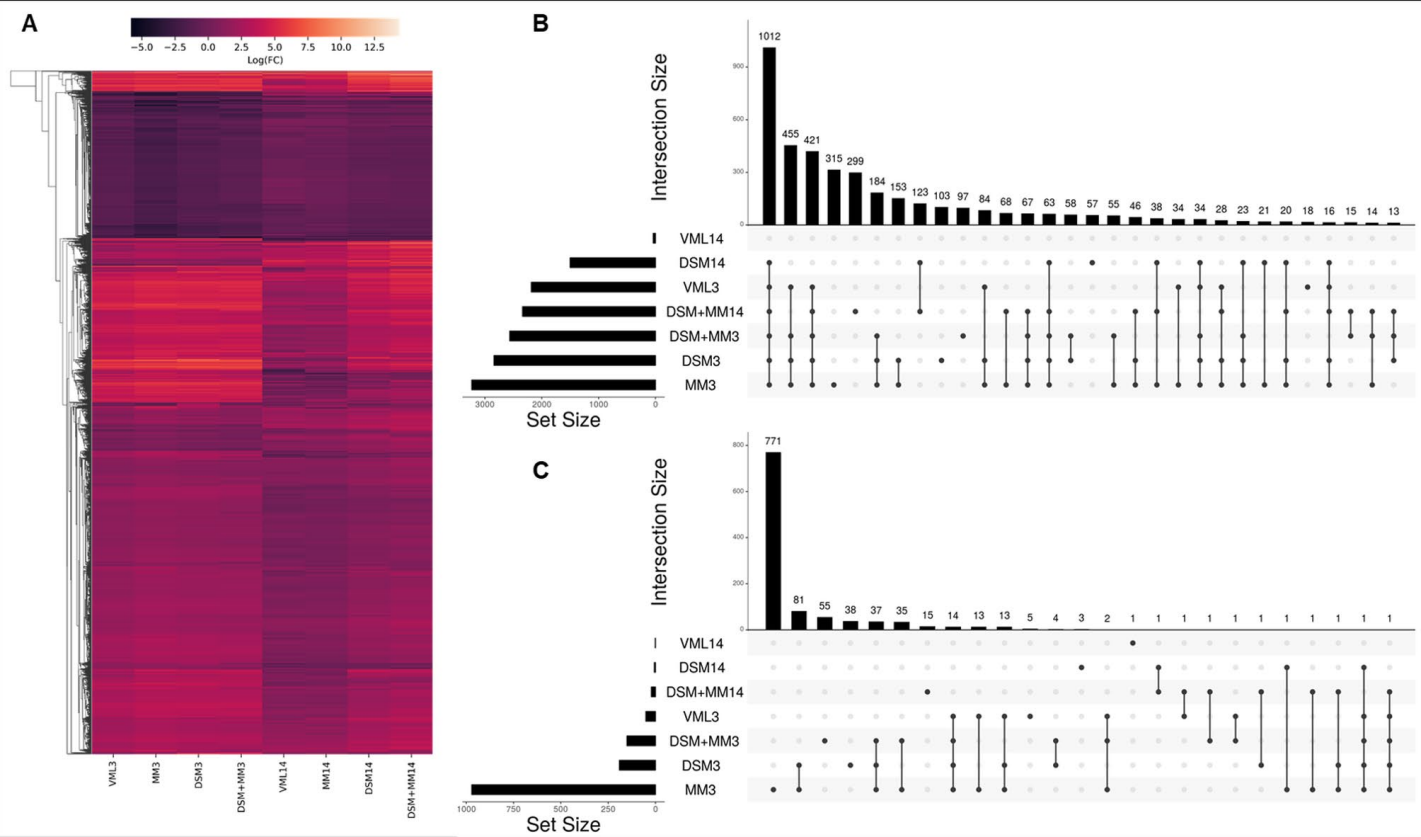
Global transcription within the VML cell community diverges profoundly based on repair strategy **(A)** Heatmap of log_2_FC for all 5174 differentially expressed genes (DEGs) across all treatment groups relative to uninjured muscle, with hierarchical clustering of gene expression data visualized using one minus Pearson correlation with average linkage. Visualization of the top 30 DEG intersections for upregulated (**B**) and downregulated (**C**) DEGs was performed using UpsetR.

**Fig. 4:**
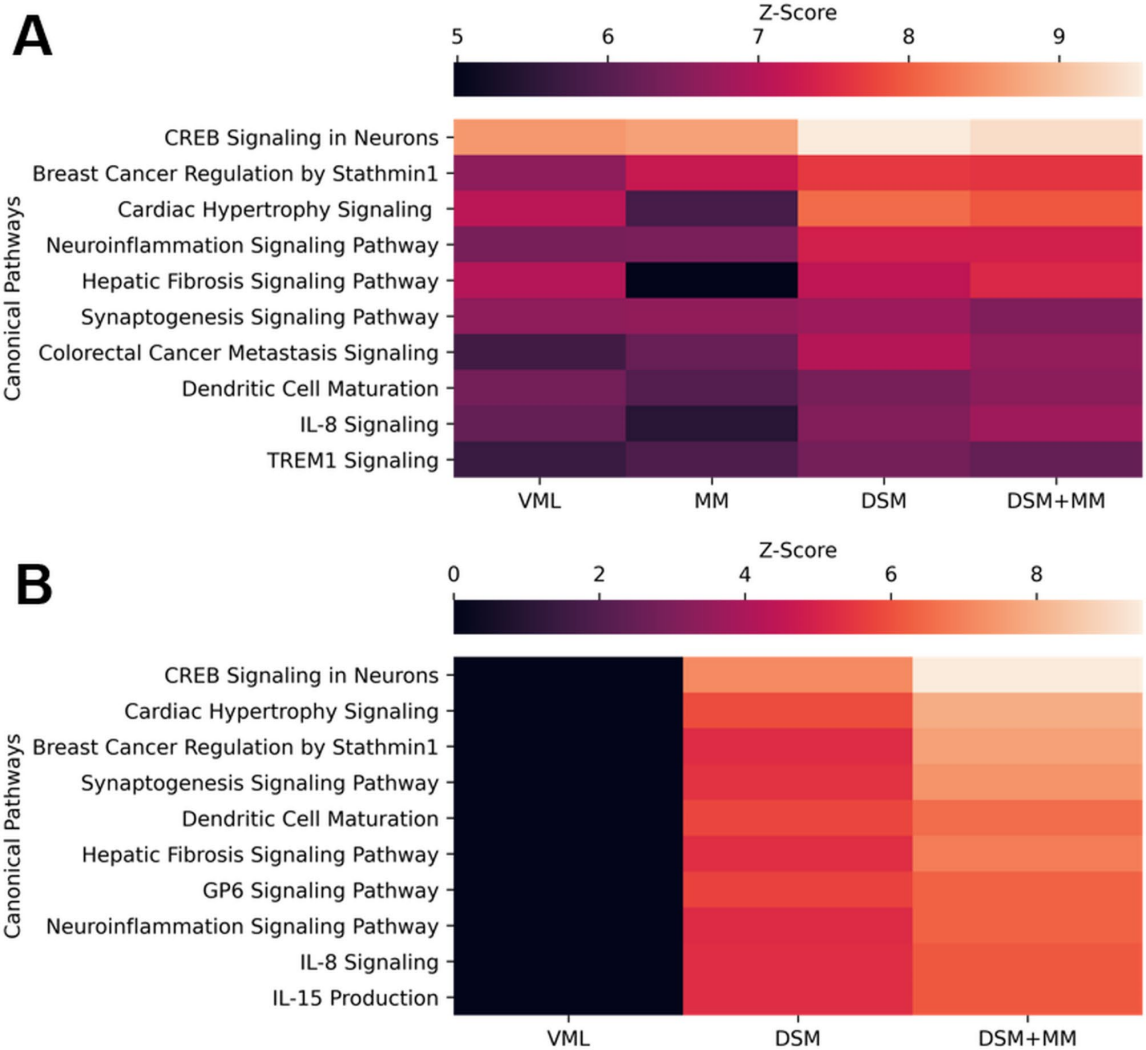
Top IPA Canonical Pathways at 3 and 14 days post injury Heatmaps of Z-score for the top 10 canonical pathways from the IPA knowledge base for treatment groups relative to uninjured muscle at (**A**) 3 days post injury and (**B**) 14 days post injury. Pathway analysis was not performed for the MM group at 14 days post injury as no genes within that group met DEG criteria

Analysis of RNA-seq data in EdgeR revealed a cumulative total of 5174 differentially expressed genes (DEGs) across all treatment groups relative to uninjured muscle (Fig. 3A). A broadly similar pattern of gene expression was observed across all groups at 3 DPI, with the notable exception of a marked downregulation of 771 genes within the MM group (Fig. 3B-C) revealed by gene ontology to be associated with diverse range of macromolecule metabolic processes (Supplemental Fig. 2). No DEGs were identified for the MM-treated group at 14 DPI (MM14). The top canonical pathway from the IPA knowledge base for groups at both time points (Fig. 4A-B**)** suggests an activation of CREB signaling which persists in the DSM and DSM + MM groups at 14 DPI and is known to promote survival of macrophages [34]. This together with the predicted activation of pathways for IL-8, TREM1, and neuroinflammation at 3 DPI as well as CREB, IL-8, IL-15, and neuroinflammation at 14 DPI suggests a general state of inflammatory signaling in all groups except MM14 and VML14. The prediction of activated hepatic fibrosis signaling likewise suggests a similar pattern reflecting general fibrosis-related transcription across the same groups.

### Myogenesis and angiogenesis transcription are generally unaffected in the early period following VML repair

**Fig. 5:**
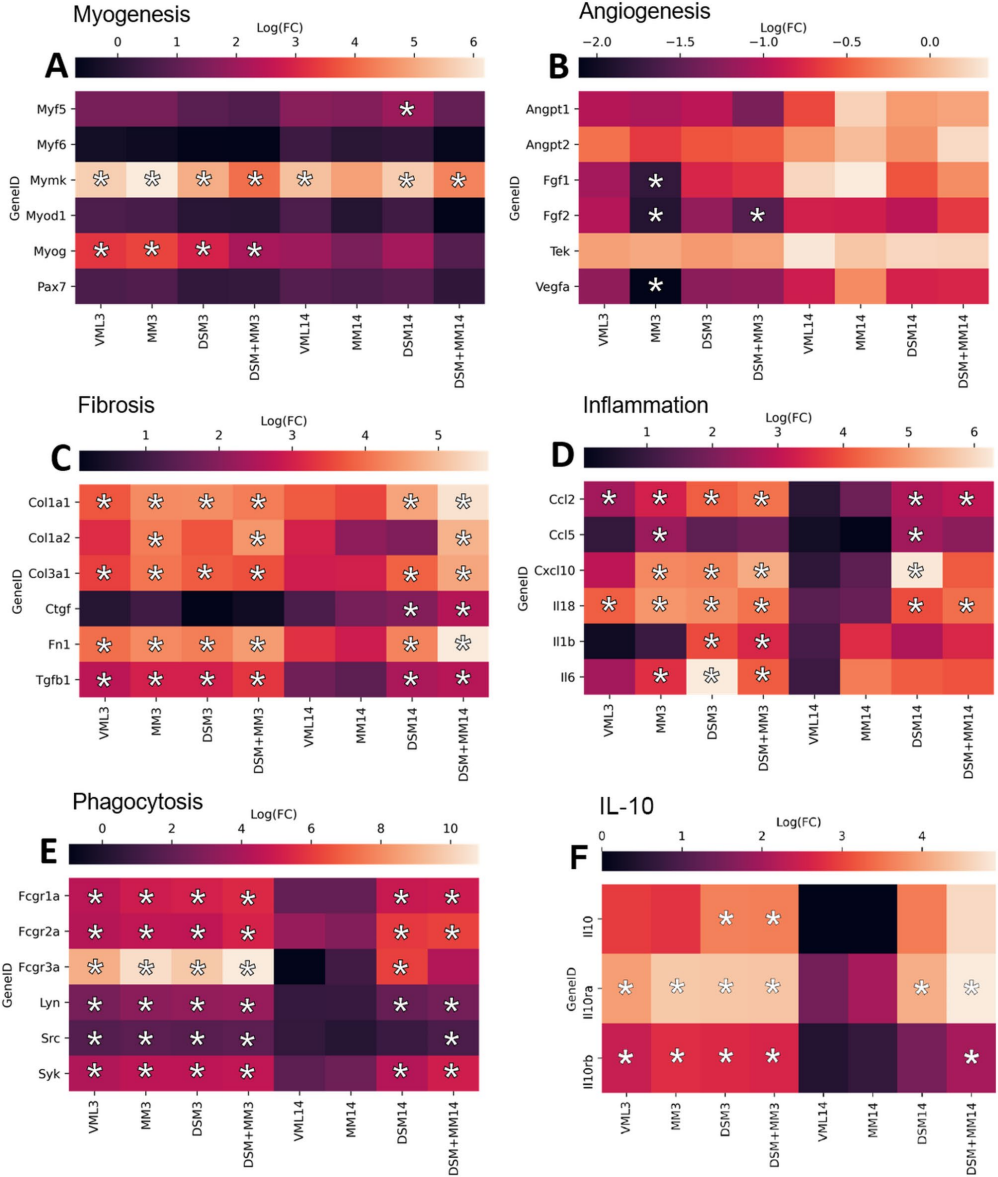
Expression of key genes for relevant biological processes Heatmaps of gene expression (log_2_FC) for all treatment groups relative to uninjured muscle for key (**A**) myogenesis, (**B**) angiogenesis, (**C**) fibrosis, (**D**) inflammation, (**E**) phagocytosis, and (**F**) IL-10 signaling related genes. Asterisks (*) indicate measurements meeting differential gene expression criteria; abs(log_2_FC > 1.5) and p < 0.05 when comparing to uninjured controls

Assessment of all groups for the classic markers of precursor cell myogenesis indicate a general lack of coordinated myogenesis signaling at 14 DPI. Notable exceptions include the upregulation of MYOG in all 3 DPI groups, the observation of upregulated MYF5 within the DSM group relative to uninjured controls at 14 DPI (p = 0.007), and significant (p < 0.05) increases in MYMK across most groups relative to control, though between-treatment differences were insubstantial. (Fig. 5A) Assessments for angiogenesis markers indicated downregulation of FGF1, FGF2, and VEGFa in MM3 group as well as downregulation of FGF2 in the DSM + MM3 group (Fig. 5B). All other groups exhibited no significant changes in the expression of angiogenesis-related transcripts. Ingenuity Pathway Analysis predicted no significant changes to myogenesis or angiogenesis at the pathway level.

### Profound upregulation of fibrosis and inflammation related transcripts persists in DSM and DSM + MM interventions at two weeks post injury

Transcripts for many well-described matrix structural constituents and enzymes, including fibrillary collagens, collagen nucleators, and matrix metalloproteinases, were found to be profoundly upregulated in both the DSM and DSM + MM groups at 14 DPI (Fig. 5C**)**, with IPA identifying significant upregulation within the hepatic fibrosis canonical pathway for each (p = 6.81E-16 with 44 DEGs and 5.63E-21 with 62 DEGs, respectively). Multiple common pro-inflammatory cytokines and chemokines were significantly upregulated in all implant groups at 3 DPI as well as the DSM group at 14 DPI, with DSM + MM at 14 DPI also exhibiting increased transcription of CCL2 and CXCL10 (Fig. 5D**).** Upregulation of the *Fcy* receptor-mediated phagocytosis pathway was further identified (p = 1.99E-12 with 27 DEGs and p = 2.35E-10 with 30 DEGs, respectively) with upregulation of transcripts for phagocytosis-activating FC receptors as well as SRC and SYK family kinases [35] identified across most groups (Fig. 5E**).** The Cell Movement category of transcripts was found by Ingenuity Pathway Analysis (IPA) to be significantly upregulated for all groups at 3 DPI and the DSM (p = 1.73E-91 with 525 DEGs) and DSM + MM (p = 2.34E-109 with 736 DEGs) groups at 14 DPI. While all 3 DPI groups exhibited patterns of transcription identified by IPA as consistent with immune cell trafficking, at 14 DPI only DSM and DSM + MM treatment continued to show such patterns (p = 5.49E-19 with 370 DEGs and p = 1.25E-21 with 510 DEGs, respectively). Of particular note, at 3 DPI, expression of the IL-10 receptor subunits IL10ra / IL10rb were significantly upregulated across all groups, however by 14 DPI expression had largely decreased and remained upregulated in the DSM + MM group only (Fig. 5F).

### Expression of key peripheral neuroregeneration genes is increased in DSM + MM repair

**Fig. 6:**
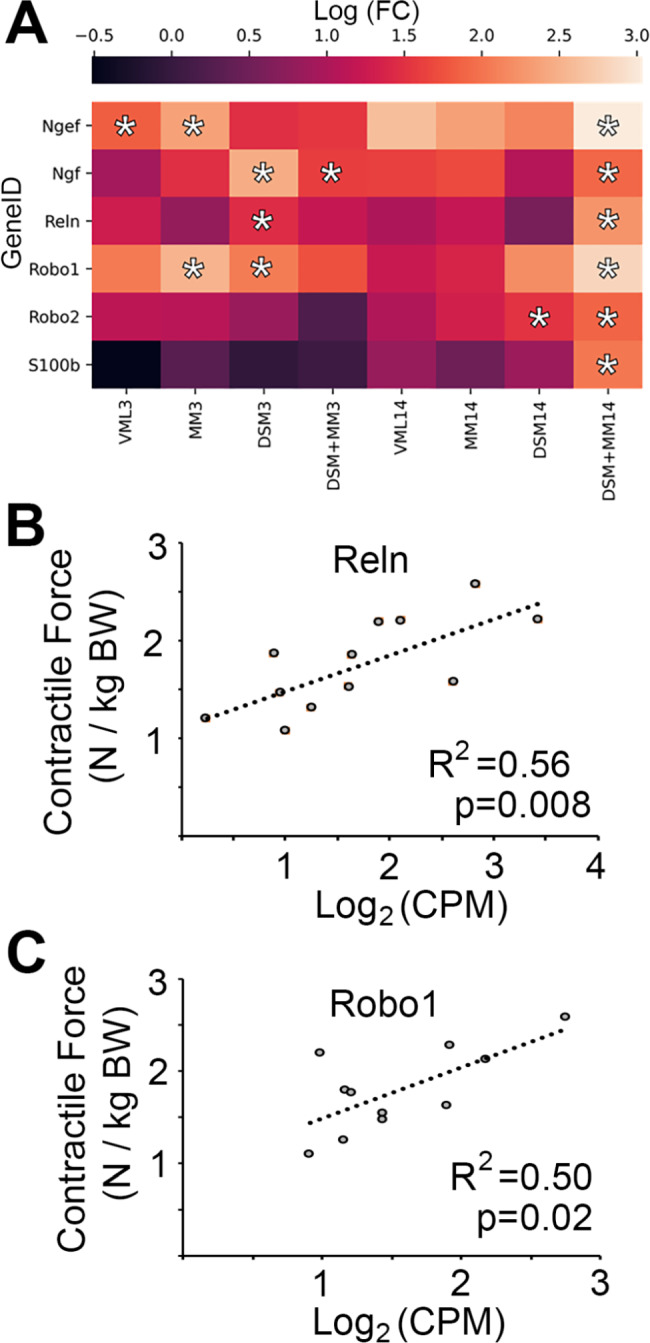
Neuritogenesis transcription is significantly increased in DSM + MM repair Heatmaps of gene expression (log_2_FC) for all treatment groups relative to uninjured muscle for key (**A**) peripheral neuritogenesis related genes. Asterisks (*) indicate measurements meeting differential gene expression criteria (abs(log_2_FC) > 1.5 and p < 0.05) when comparing to uninjured controls. (**B**) Reln (p = 0.008) and (**C**) Robo1 (p = 0.02) expression correlated moderately with animal limb torque outcomes, indicated through linear regression with R^2^ > 0.50 for each

IPA identified substantial changes in the Neuroinflammation and Axonal Guidance Signaling canonical pathways in all 3 DPI groups, with VML, MM, DSM, and DSM + MM each exhibiting ~ 80 DEGs for neuroinflammation and ~ 100 DEGs for axonal guidance. Notably, DSM and DSM + MM at 14 DPI exhibit 60 and 109 DEGs associated with axon guidance signaling, respectively, while VML and MM at 14 DPI did not exhibit such patterns; further, pathway analysis of the 299 upregulated DEGs unique to the DSM + MM group at 14 DPI indicates that 66 of the 299 are known to have roles in nervous system development and function, including neurotransmission (20 DEGs) and motor function (6 DEGs). Querying the Gene Ontology Cellular Component database for the 299 unique DEGs additionally revealed that 37 DEGs are known to localize to the projections of neurons. Querying the Gene Ontology Biological Process database indicated that 20 of the 299 DSM + MM14 unique genes have roles in synaptic signaling, and that 25 of the human homologs of the 299 DEGs have similar roles in humans. Expression levels of several genes known to have important roles in axon guidance and peripheral neuroregeneration (Ngef, Ngf, Reln, Robo1, and S100b) were found to be significantly upregulated in the DSM + MM group but not others at 14 DPI (Fig. 6A**)**. Among these genes, both Reln (p = 0.008, R^2^ = 0.56) and Robo1 (p = 0.002, R^2^ = 0.5) expression were found to correlate moderately with limb contractile force (Fig. 6B-C**)**.

## Discussion

In this work we have profiled the global transcriptomes of the VML cell community in response to several biomaterials-based repair strategies comprising DSM, MM, and a combination of the two (DSM + MM). A standard of care for VML remains elusive; MM implantation can promote some degree of repair but requires prohibitively large amounts of donor tissue for extensive VML wounds. An emerging alternative is the use of extracellular matrix scaffolds; however, many of those piloted in the literature thus far have mixed impacts on functional outcome. In our previous work the application of MM pastes to DSM prior to implantation restored half of the peak contractile force lost to VML (81% of uninjured peak contractile force vs. 62%), while other minced muscle or ECM grafts result in comparatively modest improvement to force outcomes [27]. In this study we have specifically examined transcriptomic outcomes in the early period (3–14 DPI) in order to assess whether this treatment strategy elicits transcriptional activity early in the recovery process that may explain the disparate fates of MM, DSM, and DSM + MM treatment of VML observed in long-term functional deficit [27]. We observed significant deficits among all treatment groups relative to normal muscle at 14 days post-injury. While DSM + MM treatment exhibited the highest force out of all treatment groups, the means of individual treatment groups were not significantly different from each other. It should further be noted that the in vivo limb force measurement used in this study does not preclude the effect of compensatory hypertrophy of the extensor digitorum longus on the absolute magnitude of force. These force values were measured as a routine part of the study and were not indicative of the long-term functional values since active inflammation at these early timepoints can interfere with force production.

While upregulation of some myogenesis-related transcripts relative to uninjured muscle was observed in some groups, between-treatment differences were insubstantial, in agreement with the findings of Aguilar et al. in their transcriptomic profiling of VML and MM repair [1]. The short 14-day study period, gross morphology of the muscle, and lack of significant upregulation of myogenic transcripts in DSM + MM relative to the uninjured normal condition all suggest that myogenesis is an unlikely contributor to the tissue microenvironment at this stage. Chen and Walters have suggested that muscle-derived ECM mediates force recovery in part through the provision of a physical bridge through which force can be transmitted across the site of injury [36]. Corona et al. further show that relative to untreated VML injuries, implantation of muscle ECM biases collagen deposition toward the defect site rather than intramuscular deposition and prevents reductions in fiber cross-sectional area, suggesting a protective effect of implanted muscle ECM against muscle atrophy following injury [22]. Our data indicate that the DSM component causes substantial and persistent upregulation of transcripts for the chief fibrillary collagens, collagen nucleators [37], as well as collagen-degrading matrix metalloproteinases [38], likely reflecting the remodeling of implants toward scar-like tissue as evident in Fig. 2A. This, combined with the profound upregulation of phagocytosis and cell movement related genes, illustrates a complex environment within the wound in the weeks following VML; while decellularized skeletal muscle has been shown to promote myogenesis by acting as a substrate for satellite cell migration and differentiation [39], the absence of substantive myogenic signaling suggests that myogenic processes are not substantive contributors in the early period (< 14 DPI) following VML repair, in agreement with suggestions that these processes are overwhelmed by pro-fibrotic signaling in the VML defect that is not ameliorated by repair with ECM scaffolds [7].

Implantation of cellular constructs following VML has shown the ability to promote muscle innervation as soon as a week post-repair [40], and can be augmented in the presence of exercise [17, 18]. Our observation of substantial upregulation of several neuroregeneration related transcripts suggests a possible neurologic involvement in the long-term effectiveness of DSM + MM repair on the functional outcome of VML observed in previous work [27], though significant between-treatment differences in force outcome were not observed at these early timepoints. VML injuries result in chronic motor axotomy, with denervation-induced atrophy of defect-adjacent muscle being a potential contributor to the statistically significant deficits in terminal muscle mass and contractile force observed across all groups. While most VML investigations in the literature have focused on the influence of myofiber atrophy, myogenesis, or immune involvement, Corona et al. recently demonstrated that VML results in chronic axotomy of ~ 69% of motoneurons innervating the tibialis anterior, concurrent with a large deficit in muscle force [41]. In their work it is shown that axons innervating muscle are dramatically affected by VML in a manner disproportionate to the amount of muscle removed, suggesting that axons innervating the remaining intact muscle may also be impacted. Indeed, Greising et al. suggest that attenuating pathology of the ostensibly healthy musculature adjacent to the defect may be a more fruitful target for therapeutic interventions than the prevailing focus on *de novo* myogenesis [7]. Therefore, promoting the innate ability of terminal motor axons and neuromuscular junctions to reform within defect-adjacent muscle may improve the functional outcome of VML. Further, perisynaptic Schwann cells [17] from the minced muscle graft may be better able to participate in axon repair and guidance when combined with the DSM substrate which bridges normal muscle with the wound. Though we have not observed a substantive pattern of myogenesis transcription with DSM + MM in the early period following treatment, Corona et al. have shown via GFP labeling that satellite cells sourced from MM grafts do contribute to myofiber regeneration in the longer term; the potential of other MM-resident cells to contribute to recovery from VML does not appear to have been explored. Notably, DSM exhibits a longitudinally aligned character, and a recent study found that treatment of VML with a combination of aligned scaffolds and rehabilitative exercise increases the formation of mature neuromuscular junctions [42]. The bidirectional migratory guidance provided to satellite cells by remnant “ghost” myofibers present in decellularized muscle scaffolds provides a plausible mechanism for a therapeutic effect of DSM [9]. While we demonstrate that DSM + MM repair induces the transcription of a number of key axon guidance and neuroregeneration-associated transcripts in the early period following injury, further study with histological and protein-level measures is required to better ascertain whether the aligned character of muscle matrix combined with autologous minced muscle may mediate functionally relevant nerve regeneration following VML.

Our observation of inflammatory and phagocytosis related transcription -including several chemokines- is likely a reflection of the diverse population of macrophages and other key immune cells known to infiltrate VML defects in the early period following injury. While the recruitment of macrophages as part of the acute wound response and foreign body response to implants is well described, direct participation of these cells in a variety of regenerative processes is only recently becoming better appreciated. Intriguingly, Stratton et al. demonstrated that macrophages regulate Schwann cell function during regeneration, with ablation of macrophages associated with the injury site resulting in marked reductions in conduction velocity and remyelination mediated by the absence of macrophage-derived GAS6 [43]. Cattin et al. found that macrophages assist in the formation of tissue bridges promoting the migration of repair Schwann cells during peripheral nerve regeneration [44]. Further, some initial evidence suggests that aligned scaffolds may contribute to peripheral nerve regeneration by polarizing macrophages toward a pro-regenerative phenotype, enhancing the proliferation and migration of Schwann cells. This suggests a potential role for the aligned character of DSM in mediating long term force recovery in VML [45].

We detected elevated transcription of IL-10 receptor subunits in all groups except VML and MM at 14 days post injury, yet the transcription of IL-10 only achieved significance in the DSM and DSM + MM groups at the 3-day timepoint. IL-10 is a well-established wound healing cytokine with known anti-inflammatory effects [46], and its elevated transcription may be a reflection of the population of hematopoietic cells carrying the receptor which are known to infiltrate muscle following injury. During muscle regeneration, IL-10 signaling triggers the transition of macrophages from a pro-inflammatory M1 phenotype to a pro-regenerative M2 phenotype [2, 47]. When viewed together, the IL-10 / IL10R findings suggest a potential responsiveness to IL-10 signaling at the VML repair site, but in the absence of significant endogenous IL-10 production at two weeks following intervention.

We find that the results of this transcriptomic study suggest that DSM + MM treatment promotes a pattern of transcription consistent with peripheral neuroregenerative signaling at two weeks following injury (Fig. 6). Further, when correlating to the muscle contractile force, we identified a positive relationship between the upregulation of neuritogenesis (Reln) and axonogenesis (Robo1) genes with improved force recovery. While Reln has been shown to participate in neurite outgrowth, Robo1 is known to play important role in bundling of nascent axons during muscle innervation [48, 49]. We suggest that this could potentially influence the long-term reformation of axons and corresponding neuromuscular junctions that are damaged in the defect-adjacent muscle and therefore an attenuation of denervation-induced muscle atrophy [17], though we did not assess such measures in this study. It is understood that perisynaptic Schwann cells participate in the maintenance of neuromuscular junctions [50] as well as their reestablishment following nerve injury [51]. While future study will be needed to better ascertain the functional relevance of our transcriptomic observations, we suggest that perisynaptic Schwann cells and other cell types liberated during MM preparation could participate in regeneration, and that the scaffold component provides a plausible bridge for their migration into adjacent tissue that is not present when MM is implanted in isolation. Evidence to date suggests that peripheral nerve regeneration is a process which competes with myogenesis and angiogenesis following injury, though exact regulatory mechanisms are a matter of some debate. As an example, the chemorepellent SEMA3A is secreted by satellite cells following crush injury and has been thought to prevent axon ingrowth during myofiber regeneration [52]; however, a recent study instead found a significant decrease in SEMA3A expression following injury and concluded that it is completely dispensable for reinnervation of neuromuscular junctions [53]. The increases in neuroregeneration related transcripts we have observed in our combined treatment group comes far earlier than would be expected in the typical sequence of muscle regenerative events, and could indicate early neural-myogenic crosstalk similar to that which has been documented in the context of muscle development [54]. Given that alignment of scaffold components along the major axis of scaffolds promotes neuritogenesis [55] and that aligned decellularized ECM may facilitate axon guidance [56], we suggest that a major factor in the promotion of increased neurological transcription by DSM + MM may be scaffold alignment, with the MM component providing a liberated source of muscle resident Schwann cells. Future studies in which the full community of muscle-resident cells and neuromuscular junctions in the defect-adjacent muscle are directly assessed will be needed to evaluate the role that this observed upregulation of neurological transcription may have in the long-term efficacy of DSM + MM repair.

## Conclusion

In sum, our data suggest the involvement of several key immunomodulation, neuritogenesis, axonogenesis, and axon guidance transcripts in VML repair in the early period following injury; these changes were particalular notable in the scaffold + cell treatment (DSM + MM) group. Interrogation of these and other factors influencing nerve regeneration and immune-assisted repair within injured skeletal muscle is relatively unexplored and may yield fruitful targets exploitable by a combination of drug and biomaterials-based therapies. Ultimately, VML injuries exhibit significant heterogeneity. Therefore, a range of clinical strategies incorporating appropriate biomaterials, drugs, and physical rehabilitation will be required to best address the complications of this challenging condition. As the expense of RNA-sequencing technologies decreases and the ease of downstream data analysis increases, whole transcriptome expression profiling will be an invaluable tool in guiding the development of promising therapies for the treatment of VML.

## Electronic supplementary material

Below is the link to the electronic supplementary material.

Supplementary Material 1

## Data Availability

The datasets generated during and/or analyzed in this study are available from the corresponding author on reasonable request. RNASeq data, which have been deposited in the Gene Expression Omnibus (https://www.ncbi.nlm.nih.gov/geo/query/acc.cgi?acc=GSE125896) and are fully and publicly accessible under accession number GSE125896.
