# Supplementary material for: Transcriptome profiling of a synergistic volumetric muscle loss repair strategy
Source: BMC Musculoskelet Disord. 2023 Apr 24;24:321. doi: 10.1186/s12891-023-06401-1 (PMC10124022; doi:10.1186/s12891-023-06401-1)
Supplement: Supplementary file 1 — Supplementary Material 1 [file 12891_2023_6401_MOESM1_ESM.pdf]

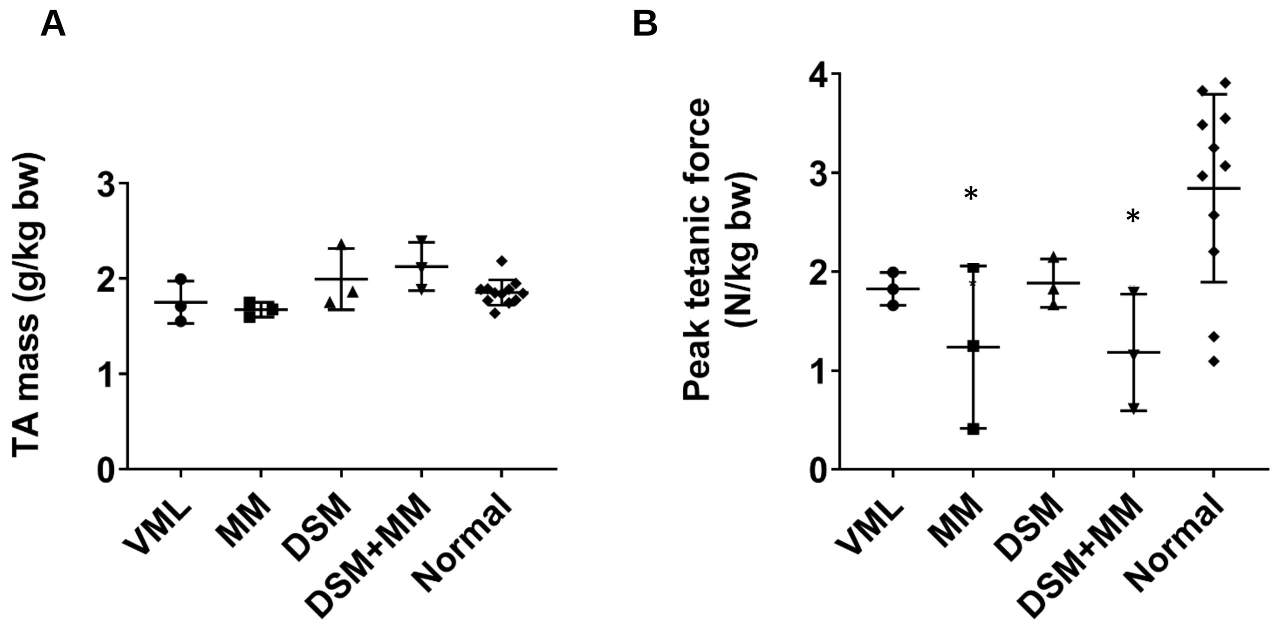

Supplemental Figure 1. Muscle mass and force at 3DPI

(**A**) Tibialis anterior mass (g/kg rat body weight) and (**B**) peak contractile force of the tibialis anterior for all groups at 3 DPI. Asterisk (*) indicate $p \leq 0.05$ when compared to normal group. Error bars are presented as ± standard deviation, with N = 3 animals per treatment group.

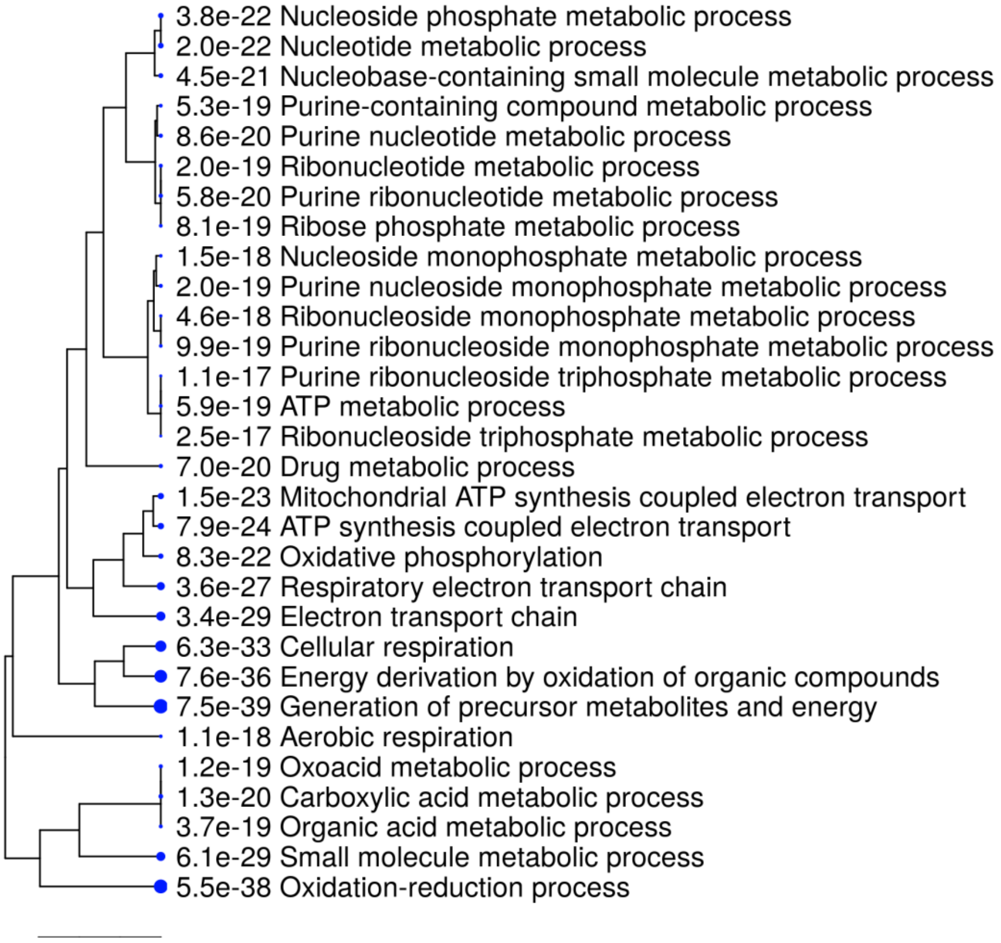

Supplemental Figure 2: Enriched pathways for uniquely downregulated genes for MM repair of VML at 3 DPI

Hierarchical clustering tree visualizing correlations of significantly enriched pathways, based on 771 uniquely downregulated DEGs for MM repair of VML at 3 days post injury (DPI) queried to the Gene Ontology Biological Process database in ShinyGO. Dot size corresponds inversely to p-value.
